# Development of an intein-mediated split–Cas9 system for gene therapy

**DOI:** 10.1093/nar/gkv601

**Published:** 2015-06-16

**Authors:** Dong-Jiunn Jeffery Truong, Karin Kühner, Ralf Kühn, Stanislas Werfel, Stefan Engelhardt, Wolfgang Wurst, Oskar Ortiz

**Affiliations:** 1 Institute of Developmental Genetics, Helmholtz Zentrum München, German Research Center for Environmental Health, Munich 85764, Germany; 2 Institute of Developmental Genetics,Technische Universität München, Freising-Weihenstephan 85354, Germany; 3 Max Delbrück Center for Molecular Medicine (MDC), Berlin 13125, Germany; 4 Institute of Pharmacology and Toxicology. Technische Universität München, Munich 80802, Germany; 5 German Center for Cardiovascular Research (DZHK), partner site Munich Heart Alliance, Munich 80802, Germany; 6 Deutsches Zentrum für Neurodegenerative Erkrankungen (DZNE), Munich 80336, Germany; 7 Munich Cluster for Systems Neurology (SyNergy) Adolf-Butenandt-Institut Ludwig-Maximilians-Universität München, Munich 81377, Germany

## Abstract

Using CRISPR/Cas9, it is possible to target virtually any gene in any organism. A major limitation to its application in gene therapy is the size of Cas9 (>4 kb), impeding its efficient delivery via recombinant adeno-associated virus (rAAV). Therefore, we developed a split–Cas9 system, bypassing the packaging limit using split-inteins. Each Cas9 half was fused to the corresponding split-intein moiety and, only upon co-expression, the intein-mediated trans-splicing occurs and the full Cas9 protein is reconstituted. We demonstrated that the nuclease activity of our split-intein system is comparable to wild-type Cas9, shown by a genome-integrated surrogate reporter and by targeting three different endogenous genes. An analogously designed split-Cas9D10A nickase version showed similar activity as Cas9D10A. Moreover, we showed that the double nick strategy increased the homologous directed recombination (HDR). In addition, we explored the possibility of delivering the repair template accommodated on the same dual-plasmid system, by transient transfection, showing an efficient HDR. Most importantly, we revealed for the first time that intein-mediated split–Cas9 can be packaged, delivered and its nuclease activity reconstituted efficiently, in cells via rAAV.

## INTRODUCTION

The discovery of the bacterial clustered regularly interspaced short palindromic repeats/CRISPR associated (CRISPR)/Cas) system is a major breakthrough in genetic engineering. Through its simplicity, it has made genome modification accessible to the wide scientific community. Cas9 is used as a generic nuclease, and a guide RNA (gRNA) confers sequence specificity on Cas9 by carrying an identical complementary sequence to a genomic region of interest (1). Nevertheless, the use of CRISPR/Cas9 as an advanced method for gene therapy has its limitations.

One of the challenges is to find an optimal delivery system that could carry all CRISPR/Cas9 components to the desired organ or cell population for genetic manipulation. As a first attempt, plasmids coding for *Streptococcus pyogenes* Cas9 (SpCas9), gRNA and a donor oligonucleotide were administered directly into the tail-vein via hydrodynamic injection. In this proof of principle, the recovery from the pathogenic phenotype of hereditary tyrosinemia, caused by a mutation in the *Fah* gene, was shown in mouse (2). Due to the low efficiency of gene correction and the method used, its translation to broader use in gene therapy would be limited to some disorders. In another attempt, adenovirus was used as the delivery system to efficiently introduce a loss-of-function mutation into the proprotein convertase subtilisin/kexin type 9 (Pcsk9) gene. This strategy would be expected to lead to decreased levels of low-density lipoprotein cholesterol and thus a reduced risk of cardiovascular diseases (3). However, the implementation of adenovirus in gene therapy can lead to a strong immune response, restricting its use to acute treatments (4). The current standard delivery system used in humans is the recombinant adeno-associated virus (rAAV), due to their high infection efficiency and very low immune response (5). Nonetheless, their packaging capacity is confined to a maximum of ∼4.7–5 kb (6,7). The human optimized SpCas9 coding sequence comprises over 4.2 kb, and, in combination with its necessary promotor sequence and gRNA, would reach a size over 5 kb, complicating the efficient production of rAAV for carrying the complete CRISPR/Cas9 system.

Various strategies to diminish SpCas9 size were followed: a) by deleting the non-essential REC2 lobe, resulting in a 133 amino acids (aa) smaller SpCas9. Albeit, the SpCas9^D175–307^ retained <50% activity of the full-length version (8); b) using smaller orthogonal SpCas9, e.g. from *Streptococcus thermophiles*, from *Neisseria meningitides* or from *Staphylococcus aureus* (9–12). However, their more complex PAM requirements make it more difficult to ﬁnd a suitable target sequence. Moreover, recent reports indicate an inverse correlation between the size of Cas9 orthologues and the complexity of the PAM sequence. This hampers the search for smaller SpCas9 orthologues with simple PAM requirements (9,10).

Recently, the structure of Cas9 has been deciphered in its apo-form and its RNA/DNA bound holo-form. This revealed the bi-lobed shaped structure of Cas9 that undergoes a large conformational re-arrangement upon binding the gRNA/DNA (8,13). The two lobes consist of a recognition lobe (REC) and a nuclease lobe (NUC). In between, there is a positively charged groove where the negatively charged nucleic acids of the holo-form reside. Structural studies render the rational engineering of Cas9 possible, either to equip it with new functionalities or to change its characteristics.

Analogous to the development of split-CreERT2 recombinase (14) and split-TALENs (15), taking advantage of the intein-extein system, it is of interest to have a precisely controllable split-Cas9, to permit the spatiotemporal control of SpCas9 activity. Post-translational regulation (e.g. by controlling the dimerization of an artificial split-enzyme) occurs within milliseconds or seconds, thus making it a precise spatiotemporally adjustable tool (16). We took advantage of SpCas9 structural knowledge and designed and created a split-intein mediated split–Cas9 trans-splicing system. This system allows the coding sequence of Cas9 to be distributed on a dual-vector system and reconstituted post-translationally.

We demonstrated the split-intein split–Cas9 functionality ﬁrst by plasmid transfection and second by administration of rAAV *in vitro*. We also showed that SpCas9D10A nickase can be split as well, reconstituting its nickase activity. In addition, we revealed that the repair template can be delivered efficiently when included in the dual-plasmid system already encoding the CRISPR/Cas9 elements.

## MATERIALS AND METHODS

### Cell Culture

The Neuro-2a cell line (ACC 148, DSMZ, Germany) was grown in complete Dulbecco's modified Eagle's medium (Gibco-BRL, Rockville, MD, USA) at 37°C and 5% CO_2_. To generate the stable surrogate reporter cell line, the same procedure published by Ortiz *et al*. (17) for M5/N9 cells was used with the following modifications: cells were co-electroporated with the linearized surrogate construct and the linearized PGK-HygR-pA for the subsequent selection against Hygromycin. After 2 weeks under selection, resistant colonies were isolated and amplified. A battery of amplified clones was co-electroporated with Cas9, crTLR#1 and a donor DNA carrying the complete Venus sequence plus 0.4 and 1.5 kb as 5′ and 3′ homology arms respectively. Forty eight hours post-transfection, the cells were analyzed by FACS and only clones with a single fluorescence signal (mVenus or TagRFP positive), indicating unique copy integration, were chosen for future experiments.

The HEK 293T reporter cell line (AAVS1 ^TLR/+^) (18) was cultivated in the same conditions as the Neuro-2a cell line.

### rAAV production and transduction

The rAAV were produced as previously described (19). In brief, HEK 293T cells were grown in triple flasks for 24 h (DMEM, 10% fetal bovine serum) before transfection with AAV plasmids. The transgene plasmids and the helper plasmid (pDP2rs, kindly provided by Jürgen Kleinschmidt, DKFZ Heidelberg) were subsequently transfected into the HEK 293T cells using Polyethylenimine (Sigma–Aldrich PEI, Polysciences). After 72 h, the viruses were purified from benzonase-treated cell crude lysates overusing an iodixanol density gradient (Optiprep, Sigma–Aldrich). AAV titers were determined by real-time polymerase chain reaction (PCR) on vector genomes using the SYBR Green Master Mix (Roche).

For transduction using AAVs, the reporter cells were cultivated in the same conditions as in the transfection experiment with the following modifications: On the transduction day, cells were given 200 μl fresh complete DMEM medium and incubated overnight with 10^6^ vg/cell. The next day, fresh medium was added and 5 days post-transduction the cells were directly analyzed by FACS.

### Transfection

X-tremGENE HP DNA transfection reagent (#06 366 244 001, Roche) was used following the provider indications. Briefly, 24 h before transfection 5 × 10^5^ cells per well were seeded in a 48-well plate. On the transfection day, cells were given 300 μl fresh complete DMEM medium and the transfection complex was prepared as follows: 0.03 μg gRNA, 0.075 μg of the different Cas9 variants expressing plasmids, 0.075 μg of the donor plasmid carrying a complete Venus cDNA (+ 0.4 and 1.5 kb as 5′ and 3′ homology arms, respectively) without promotor for traffic light reporter experiments, and completed up to one total of 0.3 μg of DNA with pSK45 plasmid per well. All experiments were done in triplicate. Twenty four hours post-transfection, fresh medium was added to the cells and 48 h post transfection the cells were directly analyzed by FACS or lysed for DNA isolation.

### FACS

Forty eight hours after the transfection, the cells were trypsinized and resuspended in a single cell suspension in ice-cold 2% fetal bovine serum (FBS) in phosphate buffered saline (PBS). A total of 10^4^ cells were counted using the FACSAria II and the number of cells with mVenus or TagRFP positive signal were expressed as a percentage of the total counted population. Cells transfected with SpCas9 plus gRNA were used as positive control and without gRNA as negative control. The data were analyzed using the BD FACSDiva Software (Version 6.1.3, BD Biosciences).

### T7 Endonuclease I assay and RFLP analysis

The following primers pairs were used to amplify the targeted loci: *Fus*, FUS-Fw (5′-CTATGGAGATGATCGACGTG-3′) and FUS-Rv (5′-TGGTTACAATTAGGGTAGTCTG-3′); *Rosa26*, Rosa26-Fw (5′-ACCTTTCTGGGAGTTCTCTGCTGCC-3′) and Rosa26-Rv (5′-TTCCCGACAAAACCGAAAATCTGTG-3′); *Rab38*, Rab38-Fw (5′- GGCCTCCAGGATGCAGACACC-3′) and Rab38-Rv (5′-CCAGCAATGTCCCAGAGCTGC-3′). In all cases the amplicons were quantified on 2100 Bioanalyzer system (Agilent Technologies). The T7 Endonuclease assay, as described previously by Reynon *et al*. (20), was used to study the gRNA-Fus and gRNA-Rosa26#1 activity. The Restriction fragment length polymorphism (RFLP) analysis was performed on the experiments with gRNA-Rosa26#3 and gRNA-Rab38#2.

### Plasmids

The same expression plasmid backbone published by Brandl *et al*. (21) was used to express the gRNA, SpCas9 and its variants. For rAAV experiments, the plasmid LITE1.0_pAAV_hSyn_CRY2PHR-NLS-VP64_2A_GFP_WPRE_bGHpA (a gift from Feng Zhang, Addgene plasmid # 48253) (22)) was used to construct the different rAAV plasmids. A CBh promoter was used to drive the expression of the split SpCas9 in the rAAV vectors (23). All plasmid and gRNAs sequences used in this study can be found in the supplementary information.

## RESULTS

### Design of Intein-mediated split SpCas9

Inteins can be easily explained as protein introns: They excise themselves out of a sequence and join the remaining flanking regions (exteins) with a peptide bond without leaving a scar (24). The coding regions of the catalytic subunit of DNA polymerase III DnaE from the cyanobacteria *Nostoc punctiforme* (*Npu*) are located in two genes, *dnaE-n* and *dnaE-c*. The *dnaE-n* encoded protein consists of an N-terminal DnaE fragment plus the N-intein, while *dnaE-c* encodes a protein that consists of the C-terminal DnaE fragment preceded by a C-Intein entity. N-intein and C-Intein recognize each other, splice themselves out and simultaneously ligate the flanking N- and C-terminal exteins resulting in the recovery of the full-length DnaE (24).

**Figure 1.**
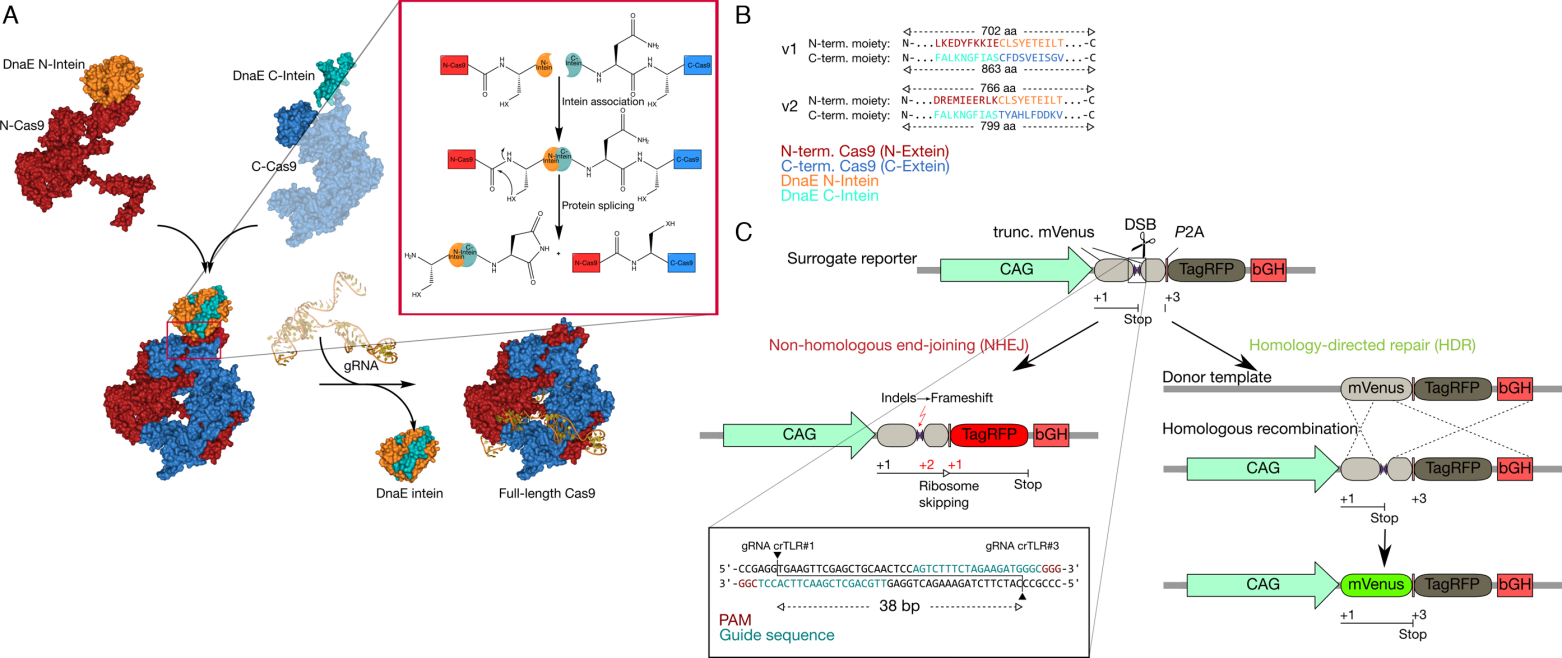
Using split-inteins for split–Cas9 reconstitution. (**A**) Upon split-intein reconstitution, the split-intein moieties splice themselves out and ligate the flanking N- and C-terminal SpCas9 halves (exteins) resulting in the recovery of active full-length SpCas9. (**B**) Split-SpCas9 version 1: DnaE N-intein (orange) is fused to the C-terminus of SpCas9^1-^^573^(N-Cas9, red), whereas DnaE C-intein (green) is fused to N-terminus of SpCas9^574-^^1368^ (C-Cas9, blue). Split-SpCas9 version 2: DnaE N-intein is fused to the C-terminus of SpCas9^1-^^637^, whereas DnaE C-intein is fused to N-terminus of SpCas9^638-^^1368^. (**C**) To measure nuclease activity, and to distinguish between HDR and NHEJ events, a traffic light reporter system was used. NHEJ events introduced by SpCas9, will often create indels that lead to frameshift mutations, resulting in the expression of TagRFP. HDR events will result in the repair of the truncated Venus by the repair template given (donor DNA; left homology arm: 0.4 kb, right homology arm: 1.4 kb) and the removal of the stop codon in the center of Venus leading to the expression of full-length Venus. Translated regions are denoted as lines under the schematic DNA with translation start site and stop codon. The box inset shows a detail of the target sequence recognized by the gRNAs crTLR#1 and crTLR#3. CAG: synthetic mammalian promoter CAG; bGH: bovine growth hormone polyadenylation site.

We use this naturally occurring phenomenon by exchanging the extein regions with the respective halves of SpCas9 (Figure 1a). The split-sites of SpCas9 were carefully chosen between Glu573 and Cys574 for the first version (v1) or between Lys637 and Thr638 for the second version (v2), since the N-terminal amino acid of the C-Cas9 in the C-Intein_C-Cas9 fusion should be Cys, Ser or Thr to ensure high splicing efficiency (7,25) (Figure 1a). Moreover, particular attention was given such that the split-sites were surface-exposed due to the sterical need for protein splicing (26).

The split–intein–Cas9 systems were created by fusing the N- or C-terminal halves of SpCas9 to the corresponding intein halves: v1 uses SpCas9^1-^^573^ and SpCas9^574-^^1368^ as split–Cas9 moieties, resulting in two fusion constructs called N-Cas9_N-Intein_v1 and C-Intein_C-Cas9_v1; v2 uses SpCas9^1-^^637^ and SpCas9^638-^^1368^ as split–Cas9 moieties, resulting in two fusion constructs called N-Cas9_N-Intein_v2 and C-Intein_C-Cas9_v2 (Figure 1b).

### Intein-mediated SpCas9 is as active as wild-type SpCas9 in surrogate reporter systems

**Figure 2.**
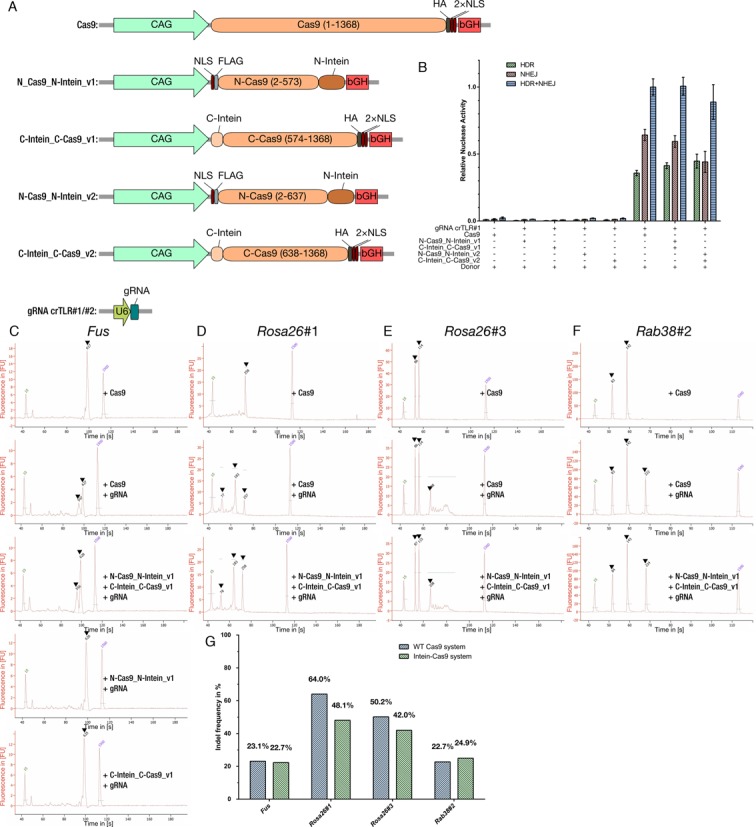
Testing split–Cas9 efficiency in Neuro-2a TLR cell lines. (**A**) Overview of the WT and split–Cas9 expression plasmids used (Cas9, N-Cas9_N-Intein_v1, C-Intein_C-Cas9_v1, N-Cas9_N-Intein_v2, C-Intein_C-Cas9_v2, gRNA crTLR#1/#2). To ensure a high expression; a strong synthetic mammalian promoter (CAG, green) and a bovine growth hormone (bGH, red) polyadenylation site was used. Cas9 cDNA is shown in orange, N-intein in dark brown and C-Intein in light brown. NLS (dark red): Nuclear localization signal. FLAG and HA tag are shown in light and dark grey respectively. For gRNA expression (turquoise), U6 promoter was chosen (dark green). (**B**) Results after FACS: only transfection with both N- and C-terminal parts of the split–intein–Cas9 system for version 1 (v1) and version 2 (v2) resulted in nuclease activity similar to wild-type SpCas9, represented in HDR or NHEJ events; transfection with only one moiety did not show any observable HDR or NHEJ events. Shown are means ± SD of three independent experiments. (**C**) The split-intein-Cas9 system v1 was used to target the fused in sarcoma (Fus) gene's second last exon. The respective segment was PCR amplified with the annotated primers for further analysis. T7 endonuclease I assay was performed after PCR on the samples to investigate the occurrence of NHEJ events. After the assay, the samples were analyzed with a Bioanalyzer. The appearance of a second band indicates the presence of indels resulting from NHEJ events. (**D**) Targeting of Rosa26 locus with the gRNA Rosa26#1. Only indels were detected when SpCas9 wild-type or both SpCas9 moieties were transfected. (**E**) Targeting of Rosa26 locus with the gRNA Rosa26#3. Indels were detected by RFLP analysis. The XbaI resistant product could be only observed when SpCas9 wild-type or both SpCas9 moieties were transfected. (**F**) Targeting of Rab38 locus with the gRNA Rab38#2. Indels were detected by RFLP analysis. The XcmI resistant product could be only observed when SpCas9 wild-type or both SpCas9 moieties were transfected. (**G**) Quantification of the nuclease activity in each target sequence. FU: fluorescence units, s: seconds.

To test if the intein-reconstituted split Cas9 is active, we used a Neuro-2a cell line carrying a single copy integrated surrogate traffic light reporter (TLR). This reporter enables the distinction between the two major repair pathways after a double-strand break, mediated by the repair machinery of the cell. This is (i) the error-prone non-homologous end-joining (NHEJ) and (ii) the high-fidelity homology-directed repair (HDR) (Figure 1c). NHEJ causes indels in the reporter region that will put TagRFP in frame in one third of the total events, allowing its expression and detection (red fluorescence); whereas HDR results in the expression of the repaired mVenus (green fluorescence) (Figure 1c). After 48 h of transient transfection with both variants of the split–intein system (N-Cas9_N-Intein and C-Intein_C-Cas9 for v1 and v2) and an U6 expressed gRNA (Figure 2a), the resulting number of fluorescent cells, corresponding to the sum of events from non-homologous end joining (NHEJ) and homology directed repair (HDR), matches the SpCas9 wild-type values, using a full-length SpCas9 and gRNA (Figure 2a and b). In contrast to that, no fluorescence signal was detected in the negative controls in which SpCas9 was expressed without gRNA (Figure 2b) or when single intein-halves of SpCas9 alone were co-expressed with the gRNA (Figure 2b).

### Targeting efficiency of endogenous genes with intein-mediated split–Cas9 system is comparable to wild-type SpCas9

In subsequent experiments, we validated our Intein-Cas9 system in several endogenous loci (*Fus, Rosa26* and *Rab38*) in Neuro-2a cells. The first gene chosen was *Fus* (fused in sarcoma) because of its importance as a model for amyotrophic lateral sclerosis (27). The DNA of the entire cell population was isolated 48 h after the transient transfection. The region containing the CRISPR/Cas9 target site was PCR-amplified and the T7 endonuclease I assay was performed. Indels will form mismatched duplexes after the denaturing and reannealing step. T7 endonuclease I cleaves these mismatched regions and the existence of indels was revealed by the presence of digested PCR products (Figure 2c). As expected, only samples from cells exposed to the SpCas9, or to both moieties, presented the predicted digestion products, showing both a similar percentage of indels (23.1 and 22.7%, respectively), while in the negative controls none were detected (Figure 2c and g).

To validate the results obtained targeting the *Fus* gene, two other gene loci were targeted. We targeted *Rosa26* as a second genomic locus with two different gRNAs (Figure 2d and e). *Rosa26* is widely used as a safe locus in mouse to knock in foreign DNA (28). As in the previous experiment, the region containing the predicted target sequence was amplified by PCR. The presence of indels when gRNA-Rosa26#1 was used was revealed by the T7 endonuclease I assay, and the existence of an XbaI site on the binding site of gRNA-Rosa26#3 permitted us to analyze the existence of indels by RFLP. When gRNA-Rosa26#1 was used, the expected digested products were only detected when SpCas9 or the two SpCas9 moieties were added (64.0 and 48.1%, respectively) (Figure 2d, g). These data were confirmed using a second gRNA, gRNA-Rosa26#3. In this case, the indels will disrupt the XbaI site, resulting in XbaI resistant mutant DNA fragments (Figure 2e). Again we observed that the nuclease activity observed with SpCas9 intein-split version (42.9%) was comparable to wild-type SpCas9 (50.2%) (Figure 2e and g).

As a third locus, we targeted the *Rab38* locus (Figure 2f). This locus was used by us before as a proof of principle genomic region to test nuclease activity of ZFN, TALEN and CRISPR/Cas9 (21,29). The region containing the target sequence of the gRNA-Rab38#2 was PCR-amplified and analyzed by RFLP. The presence of indels was revealed by loss of XcmI site, whereas the wild-type DNA was digested (Figure 2f). The undigested product was only detectable when both split SpCas9 moieties (24.9%) or SpCas9 (22.7%) were used (Figure 2f and g).

### The intein-mediated split–Cas9 system can be efficiently delivered via rAAV

**Figure 3.**
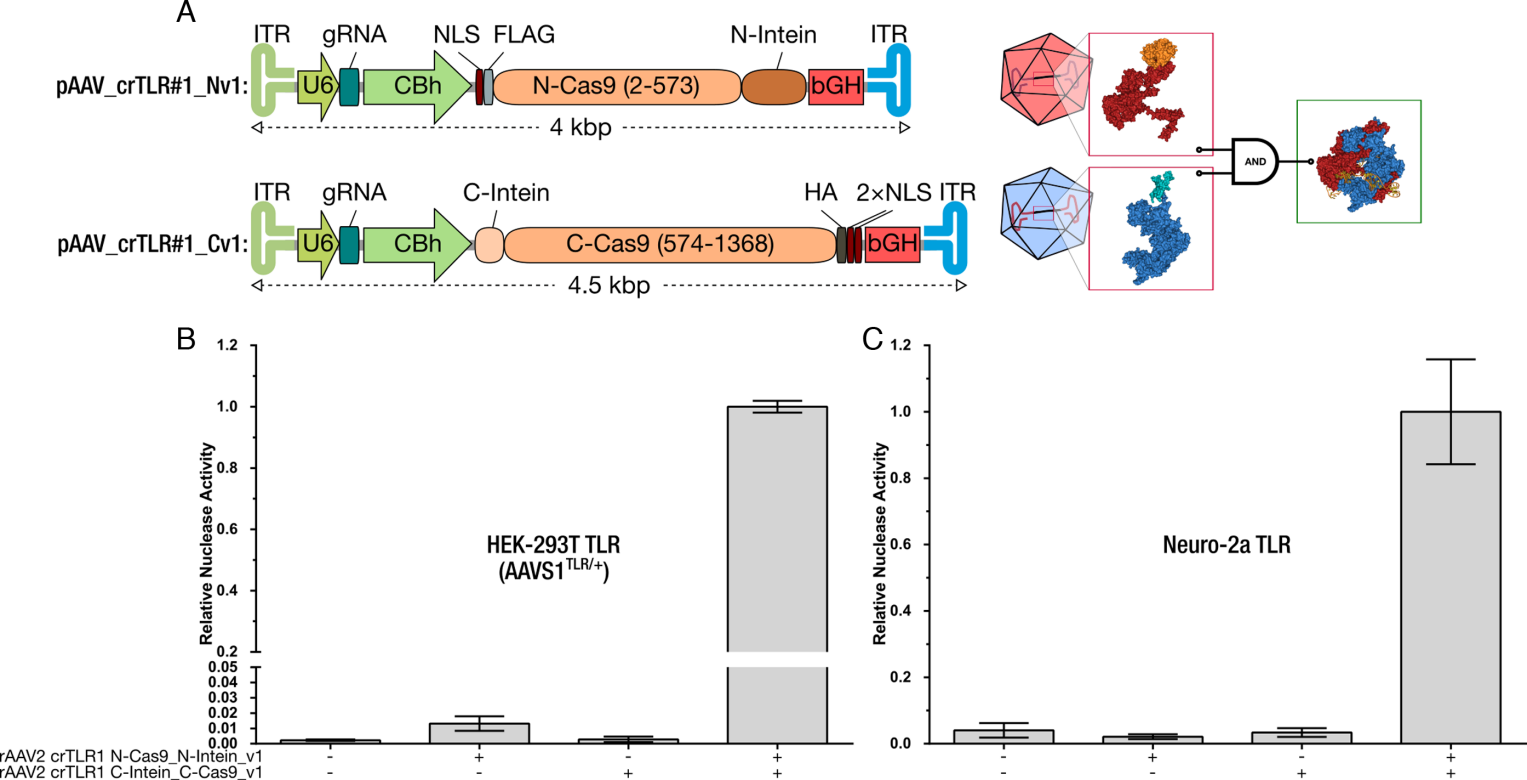
Demonstration that the split-intein split-SpCas9 system can be delivered by rAAV. (**A**) Overview of the split–Cas9 rAAV constructs (pAAV_crTLR#1_Nv1, pAAV_crTLR#1_Cv1). To ensure a high expression; a strong synthetic mammalian promoter (CBh, green) and a bovine growth hormone (bGH, red) polyadenylation site was used. Split Cas9 cDNA is shown in orange, N-intein in dark brown and C-Intein in light brown. NLS (dark red): Nuclear localization signal. FLAG and HA tag are shown in light and dark grey, respectively. For gRNA expression (turquoise), U6 promoter was chosen (dark green). The inverted terminal repeats (ITR) are shown in light blue and light green. (**B** and **C**) Only nuclease activity was detectable when the two rAAV carrying the two moieties were added to the AAVS1 ^TLR/+^ (b) or to the Neuro-2a TLR cells (c). In the experiments with only one of the moieties, the nuclease activity was indistinguishable from the negative control. Shown are means ± SD of three independent experiments.

The goal of developing an intein-mediated split–Cas9 system was to permit the efficient delivery via rAAVs. To demonstrate this, two recombinant AAV were produced, each carrying a split-half of the system (pAAV_crTLR#1_Nv1 and pAAV_crTLR#1_Cv1) (Figure 3a). A human cell line, AAVS1^TLR/+^ (18), and a mouse cell line, Neuro-2a TLR, were used to validate our system. When the AAVS1 ^TLR/+^ cell line was transduced with both rAAVs, the relative nuclease activity was at two orders of magnitude higher than the negative control or when only one rAAV was used (Figure 3b). In the case of the Neuro-2a TLR, a similar result was observed. The relative nuclease activity was one order of magnitude higher than the negative control and the single rAAV experiments (Figure 3c). In both cases, the actual activity observed was even higher because, with this reporter system, only one-third of the theoretical total nuclease activity is detected. These results demonstrate that the delivery of a complete full CRISPR/Cas9 system is possible using two rAAV without being restrained by truncated elements.

### An intein-mediated Cas9 D10A nickase is functional and comparable to SpCas9 D10A

**Figure 4.**
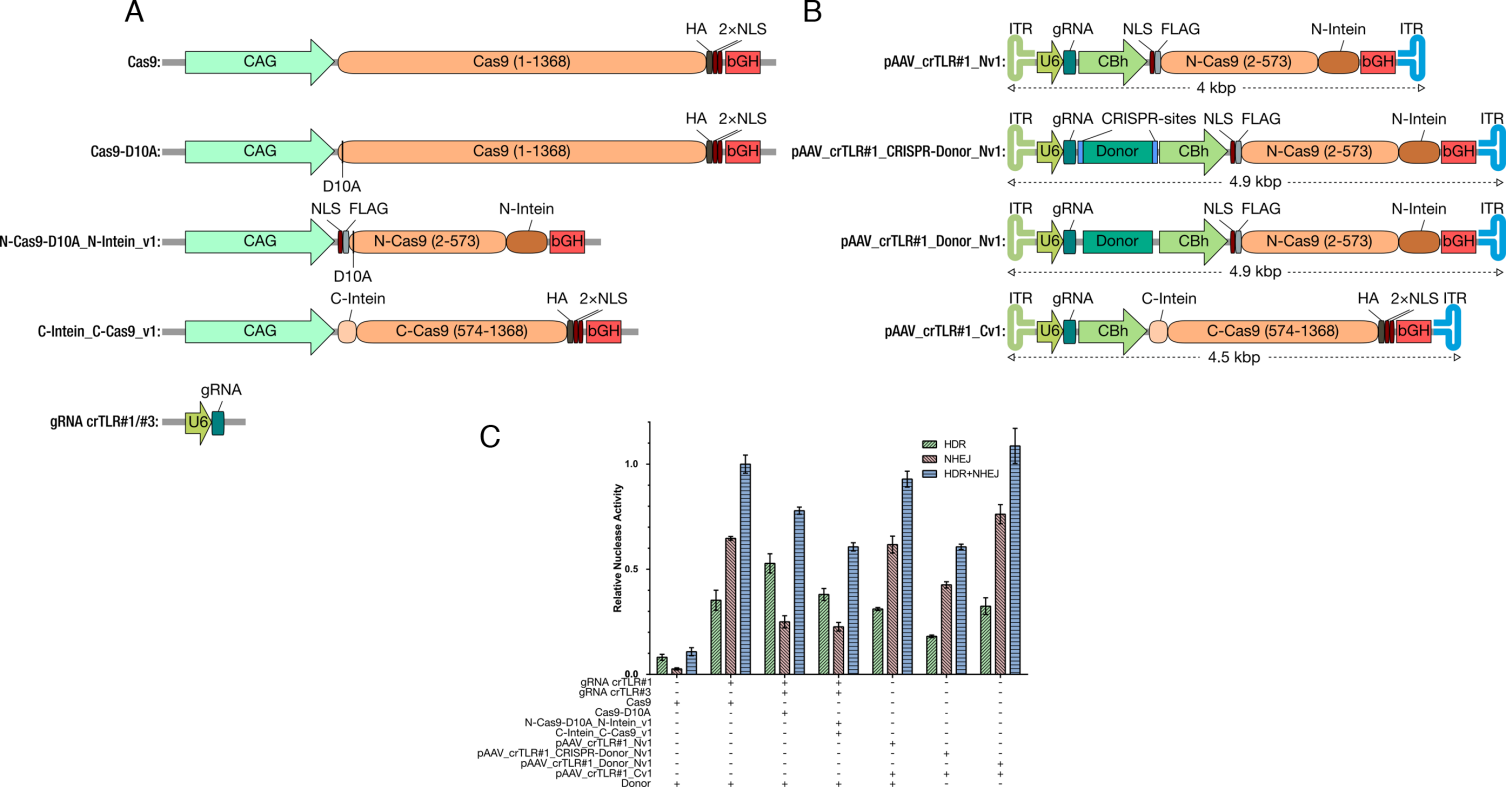
Plasmid transfection experiment for comparison of SpCas9 wild-type and nickase with its respective split versions, and of donor DNA as separate plasmid or accommodated directly on the AAV plasmid. (**A**) Overview of the split–Cas9 plasmids used to express SpCas9, SpCas9D10A and its split version (Cas9, Cas9D10A, N-Cas9_N-Intein_v1, C-Intein_C-Cas9_v1, gRNA crTLR#1/#2. (**B**) rAAV plasmids used: without donor sequence (pAAV_crTLR#1_Nv1), carrying the donor DNA flanked by CRISPR sites (pAAV_crTLR#1_CRISPR-Donor_Nv1) or not flanked (pAAV_crTLR#1_Donor_Nv1), C-Cas9 expression plasmid (pAAV_crTLR#1_Cv1). CBh promoter is shown in green, bovine growth hormone (bGH, red). Split Cas9 cDNA is shown in orange, N-intein in dark brown and C-Intein in light brown. NLS (dark red): Nuclear localization signal. FLAG and HA tag are shown in light and dark grey, respectively. For gRNA expression (turquoise), U6 promoter was chosen (dark green). The inverted terminal repeats (ITR) are shown in light blue and light green. Donor DNA sequence (Donor, dark green) (**C**) The double nicking strategy, SpCas9D10A in combination with two gRNAs, showed a preference for HDR compared to wild-type. This effect was also observed with split-SpCas9D10A but with decreased activity. With the different DNA donor strategies no differences were observed, but with donor DNA flanked by CRISPR sites reduced HDR was observed. Shown are means ± SD of three independent experiments.

SpCas9 D10A nickases have been reported to significantly reduce genomic off-targets *in vitro*, which is of great importance for the usage of CRISPR-Cas9 in gene therapy (30). Therefore, we designed an intein-mediated split–Cas9 system (Figure 4a) to compare its efficiency in HDR-NHEJ-ratio to the unmodified SpCas9D10A nickase using the traffic light reporter cell line and two gRNAs (crTLR#1 and crTLR#3) (Figure 1c) and to the SpCas9 wild-type. Interestingly, the nickases showed higher preference for HDR than the wild-type SpCas9 (Figure 4c) with reduced NHEJ values. The split-nickase also shares the same ratio HDR versus NHEJ than SpCas9D10A nickase, but with a slightly overall decreased activity compared to wild-type. Its HDR efficiency was nevertheless as efficient as wild-type SpCas9 but with greatly reduced NHEJ (Figure 4c).

### The donor DNA can be accommodated on the same plasmid encoding the split–CRISPR/Cas9 system

Many inherited diseases are monogenetic, originating from a point mutation in the corresponding gene, which therefore can be, in theory, corrected if a template region is provided after a DSB event. There is about 0.9 kb left on the dual-vector system before reaching the 5 kb, to integrate additional sequences. Thus, we included a donor sequence into one of the split–Cas9 plasmids (Figure 4b), and tested their efficiency using our TLR reporter system, in comparison to adding the donor as an independent plasmid. In addition, two versions of the rAAV-vector-encoded repair template were created: the first version contained the donor region, while, in the second version, the donor region was additionally flanked by CRISPR recognition sites (pAAV_crTLR#1_CRISPR-Donor_Nv1, pAAV_crTLR#1_Donor_Nv1, Figure 4b). When this system was tested by plasmid transfection, the HDR values observed with the non-flanked donor were comparable to the wild-type and split SpCas9 variants in the presence of an extra donor plasmid (Figure 4c). In the case of the flanked donor, the nuclease activity was lower. This could be probably caused by DNA instability after being cut by SpCas9 (Figure 4c). These results can be useful in designing a vector system for genome engineering with a lower number of plasmids, making the transfection of all of them easier.

## DISCUSSION

CRISPR/Cas9 has been widely adopted as a powerful tool for gene targeting in several different species and, more recently, for *ex vivo* gene therapy treatment. The B2M and CCR5 receptors in human hematopoietic stem and progenitor cells were successfully knocked out, as recently shown by Mandal *et al*. (31). However, in spite of its immense potential, use of the CRISPR/Cas9 system for *in vivo* gene therapy is impeded by the difficulties in ensuring efficient delivery. Recently, successful delivery of SpCas9 by rAAVs has been published (32). Nevertheless, this was achieved by strict limitations in the Cas9 expression promoter length. If longer promoters are needed to drive the Cas9 expression to a specific cell population, an alternative strategy is needed. Thus, there exists a pressing need to develop alternative systems to decrease the size to be carried.

Here, we showed that SpCas9 can be functionally split and rapidly reconstituted out of two inactive split-fragments using *Npu* split-inteins, without impairing its endonuclease efficiency. We also show that rAAV vectors are an amenable delivery system for split SpCas9, offering new possibilities for gene therapy. Splitting proteins is not always trivial because split proteins tend to form aggregates when their hydrophobic core regions are exposed to solvent when split (33). In addition, folding order in some cases is crucial when protein subdomains need to fold in a cooperative manner (34). In contrast, the bilobed spatial tertiary structure of SpCas9, and its hydrophilic and positively charged groove between the two lobes in which the gRNA/DNA partially resides, appears ideal for splitting (8).

In this work, we prove the feasibility of a split–Cas9 system. Using this strategy, it is possible to circumvent the packaging limit of rAAVs for gene therapy applications, by generating two different rAAV populations encoding each of the respective halves of Cas9 fused to the corresponding inteins (7). Furthermore, this strategy could be also used for intersectional mutagenesis to improve specificity by using different promoters. Only cells that co-express both moieties lead to the reconstitution of an active SpCas9, leading to a predicted improved accuracy in cell targeting in *in vivo* applications. Another improvement of the system would be the replacement of the naturally occurring split-inteins of *Npu* with the artificial VMA split-intein from *S. cerevisiae* (*Sce*) (35). In contrast to the *Npu* DnaE intein system, the *Sce* VMA split-intein pair is artificially split and shows no affinity between its halves. This allows conditional *in vivo* reconstitution of the artificial Sce split-intein and thus the reconstitution of full-length Cas9 by protein pairs that interact with each other upon certain chemical or physical triggers, e.g. FKBP/FRB for ligand-induced dimerization (35,36) or Cry2/Cib1 and PhyB/PIF6 for a light-induced dimerization (36).

We also showed that converting the intein-nuclease system into a nickase system was analogously possible. Of note, we observed that, with the double nicking strategy, the HDR is preferred over NHEJ as a DNA repair mechanism. Increasing HDR makes the double nicking system a very attractive option for gene repair-based therapeutic strategies. In addition, we demonstrated that it is not necessary to deliver the repair template as an extra plasmid, opening the possibility to accommodate DNA templates on the same dual rAAV vector system. Recently, a similar approach was published, where a plasmid, carrying as donor DNA the entire CRISPR/Cas9 system sequence flanked by homology arms, was correctly integrated in a pre-defined site (37). However, Gantz *et al*. focused on the generation of germline mutations in *drosophila melanogaster*. In this report, we showed that also gene repair is efficiently possible in a mouse cell line. This emphasizes its potential utility for future gene therapy strategy design. The same principle can be also applied for the nuclease-dead SpCas9 (dCas9) fused to various transcription activators/repressors or epigenetic modulators (38,39) as well as for RNA targeting (40), and developing new genetic circuits using logic gates (41,42).

During the review process of this manuscript, two independent groups published data showing that it is possible to split SpCas9 by using different strategies (43,44). Zetsche *et al*. (43), using a rapamycin-inducible split–Cas9 system, also described that one of the most active split-sites was the same we identified in our split-intein split–Cas9 system (Glu573–Cys574). Nevertheless, Zetsche used a lentiviral system that stably integrated the rapamycin-inducible expression cassettes into the genome. Afterwards, they selected cells carrying these constructs and achieved the maximum efficiency (40%) after 4–6 weeks of rapamycin incubation (43). Most recently, Wright *et al*. (44) also showed that split–Cas9 is functional in *in vitro* assays. This was shown by *in vitro* cleavage of dsDNA via the pre-assembled recombinant produced halves of split–Cas9 when gRNAs were present. In spite of this, upon nucleofection of the pre-assembled complex into cells, no notable nuclease activity was observed (0.6%). In contrast, wild-type SpCas9 showed 22% genomic modifications.

In summary, we were able to prove that it is possible to split SpCas9 and its nickase derivatives using natural split–inteins without affecting its endonuclease activity. Furthermore, we demonstrated for the first time that the split SpCas9 can be efficiently delivered using a dual rAAV vector system. Moreover, we showed that the delivery of a repair template on the dual-vector system is possible. This approach allows the exploitation of the CRISPR/Cas system potential for gene therapy, avoiding the existing rAAV packaging size problem.

## SUPPLEMENTARY DATA

Supplementary Data are available at NAR Online.

SUPPLEMENTARY DATA
