# Supplementary material for: Development of an intein-mediated split–Cas9 system for gene therapy
Source: Nucleic Acids Res. 2015 Jun 16;43(13):6450–8. doi: 10.1093/nar/gkv601 (PMC4513872; doi:10.1093/nar/gkv601)
Supplement: SUPPLEMENTARY DATA [file supp_43_13_6450__index.html]

Development of an intein-mediated split–Cas9 system for gene therapy — SUPPLEMENTARY DATA 

# Development of an intein-mediated split–Cas9 system for gene therapy

## SUPPLEMENTARY DATA

- SUPPLEMENTARY DATA
